# Biaxial tensile tests identify epidermis and hypodermis as the main structural elements of sweet cherry skin

**DOI:** 10.1093/aobpla/plu019

**Published:** 2014-04-11

**Authors:** Martin Brüggenwirth, Heiko Fricke, Moritz Knoche

**Affiliations:** Institute for Horticultural Production Systems, Fruit Science Section, Leibniz University Hannover, Herrenhäuser Straße 2, 30419 Hannover, Germany

**Keywords:** Biomechanics, fracture, mechanical properties, *Prunus avium*, rheology, skin, strain, stiffness.

## Abstract

The skin of developing soft and fleshy fruit is subjected to considerable growth stress, and failure of the skin is associated with impaired barrier properties in water transport and pathogen defence. The objectives were to establish a standardized, biaxial tensile test of the skin of soft and fleshy fruit and to use it to characterize and quantify mechanical properties of the sweet cherry (*Prunus avium*) fruit skin as a model. A segment of the exocarp (ES) comprising cuticle, epidermis, hypodermis and adhering flesh was mounted in the elastometer such that the *in vivo* strain was maintained. The ES was pressurized from the inner surface and the pressure and extent of associated bulging were recorded. Pressure : strain responses were almost linear up to the point of fracture, indicating that the modulus of elasticity was nearly constant. Abrading the cuticle decreased the fracture strain but had no effect on the fracture pressure. When pressure was held constant, bulging of the ES continued to increase. Strain relaxation upon releasing the pressure was complete and depended on time. Strains in longitudinal and latitudinal directions on the bulging ES did not differ significantly. Exocarp segments that released their *in vivo* strain before the test had higher fracture strains and lower moduli of elasticity. The results demonstrate that the cherry skin is isotropic in the tangential plane and exhibits elastic and viscoelastic behaviour. The epidermis and hypodermis, but not the cuticle, represent the structural ‘backbone’ in a cherry skin. This test is useful in quantifying the mechanical properties of soft and fleshy fruit of a range of species under standardized conditions.

## Abstract (precis)

Sweet cherry and other fleshy fruit crack when the surface is exposed to water. Osmotic water uptake is believed to increase fruit volume and hence surface area, thereby subjecting the skin to stress and strain. The objectives were to (1) establish a standardized biaxial tensile test that simulates the in vivo strain of the skin and (2) characterize its mechanical properties. A bulging device was used to pressurize skin segments. Pressure and extent of bulging were monitored. The data demonstrate that (1) epidermis and hypodermis form the structural backbone of the fruit skin and (2) deformation is reversible.

## Introduction

A fruit's skin is a complex tissue comprising the polymeric cuticle, a layer of epidermal cells and, in most soft-fruit species, one or several layers of hypodermal cells. Fruit are unlike most other plant organs. Throughout their development, the skin is subjected to continuous strain due to growth (Skene 1980; Grimm *et al.* 2012).

Strain-induced skin failure severely compromises the skin's function as a barrier to the ingress of pathogens and to the egress of water. For a soft, fleshy fruit, skin failure also limits its mechanical role as a structural ‘shell’ for the developing ‘insides’. From the perspective of commercial horticulture, fruit skin failure is associated with greatly reduced crop quality and thus value. Prominent examples include the rain-cracking of many stone and berry fruit and the russeting of many pome fruit.

In the last two decades, a considerable number of studies have focused on the mechanical properties of fruit skins. Many of these employed uniaxial tensile tests of isolated cuticles (for recent reviews, see Dominguez *et al.* 2011*a*, *b*), whereas only a few used entire skin composites (Matas *et al.* 2004; Khanal *et al.* 2013*b*). In uniaxial tests, a specimen, usually a rectangular or dumbbell-shaped strip of cuticle or skin, is subjected to a defined force applied along the axis of extension and the force and extension are monitored. The force/extension (uniaxial strain) relationships so established are analysed.

The first, and only, biaxial test of fruit skin was published by Bargel *et al.* (2004) who reported a biaxial tensile test to compare the properties of cherry skin and polyethylene films. Bargel *et al.* (2004) pressurized circular skin samples from below and monitored the extent of bulging. In this biaxial test, the skin sample is subjected to force vectors oriented in radial directions—as the spokes of a wheel. Because the bulging of the skin is associated with an increase in area, a pressure/area extension (biaxial strain) relationship is established in a biaxial tensile test. This biaxial tensile test offers a number of important advantages over uniaxial testing. First, biaxial tests reflect the natural growth stresses occurring in roughly spherical organs such as fruits. Second, depending on the mechanical properties of the tissues and the geometry of the specimen, uniaxial tensile tests result in a considerable narrowing of the specimen during its extension. This can be easily visualized when stretching a piece of woven fabric. Extensions in the directions of the warp or weft are less than those ‘on the bias’ (i.e. at 45° to the thread directions). In the latter case, a uniaxial extension is accompanied by a significant narrowing. This narrowing yields a severe overestimation of strain and a severe underestimation of the modulus of elasticity (Niklas 1992). These arguments make the approach by Bargel *et al.* (2004) particularly interesting. However, two important findings have been reported since, which may affect the data and its interpretation. First, the skin of a cherry is markedly strained *in situ* and this strain is rapidly released upon excision (Grimm *et al.* 2012). The skin's *in situ* strain is up to 36.0 % (Grimm *et al.* 2012). Maintaining this level of strain, *ex situ* (i.e. in an excised segment), requires special arrangements to be made. Second, exposing excised skin samples to water causes uncontrolled water uptake and bursting of cells (Simon 1977), which in turn is likely to affect the mechanical properties of the skin sample that it is desired to measure (assuming of course that the latter reside with the cellular components). The consequences of these findings for the reported rheological properties of the skin are unknown.

The objectives of our study therefore were (i) to compare the inferred mechanical properties of cherry skin using uniaxial and biaxial tensile tests, (ii) to establish a standardized test system and protocol for biaxial tensile testing of fruit skin, and (iii) to characterize and quantify the rheological properties of cherry skin using this protocol.

## Methods

### Plant material

Mature sweet cherries ‘Adriana’, ‘Burlat’, ‘Merchant’, ‘NY242’, ‘Rainier’, ‘Regina’ and ‘Samba’ and sour cherries (*Prunus cerasus*) ‘Morellenfeuer’ and ‘Ungarische Traubige’ were obtained from glasshouse-grown or field-grown trees grafted on ‘Gisela 5’ rootstocks (*P. cerasus* × *P. canescens*) except for ‘Morellenfeuer’ (rootstock ‘Maxma Delbard’, *Prunus avium* × *P. mahaleb*). European plums (*Prunus* × *domestica*; ‘Hanita’, ‘Nancy’ and ‘Wangenheim’ grafted on ‘Wangenheimer’, ‘Jaspe Fereley’ and ‘St Julien’ rootstocks, respectively) were all field grown. Trees were cultivated at the experimental station of Leibniz University, Hannover (long. 9°49′E, lat. 52°14′N) or, in case of sweet cherry ‘NY242’, at the Esteburg research station, Jork (long. 9°40′E, lat. 53°31′N). Grapes (*Vitis vinifera*; ‘Fanny’, ‘Nero’) and cape gooseberries (*Physalis peruviana*) were purchased locally. For the latter species, cultivar and picking date were unknown. Fruit were selected for uniformity of maturity and size on the basis of colour and mass, and lack of visual defects. Almost all experiments were conducted using fruit picked on the same day. The only exception were the experiments on the effect of orifice diameter, thickness of the fruit skin samples and the effects of pectinase treatment, where fruit were placed in a cold room (2 °C, 95 % relative humidity) and examined within 36 h of harvest.

### Elastometer

**Figure 1.**
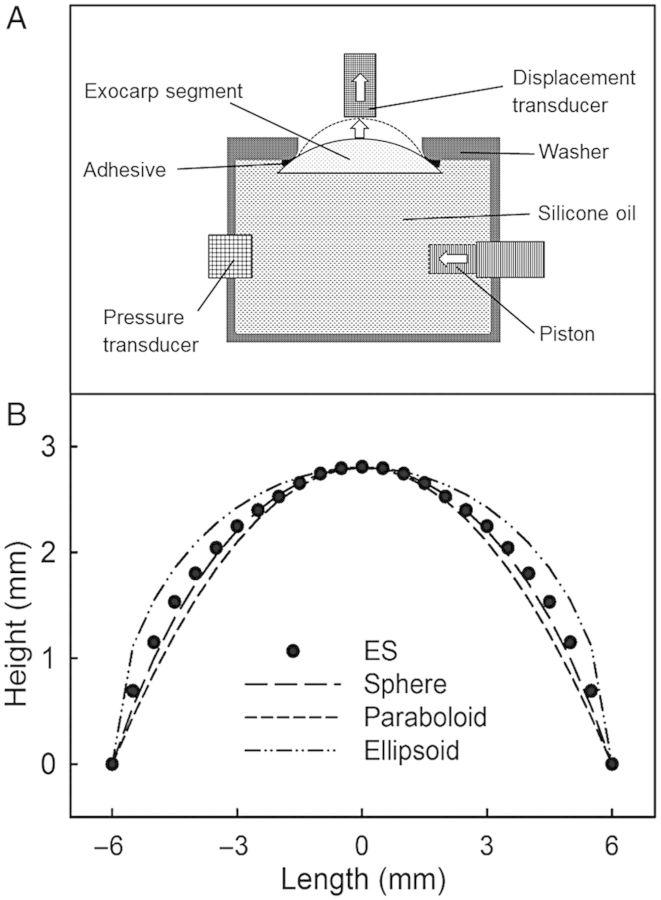
(A) Schematic drawing of the elastometer used for biaxial tensile testing. A hydrostatic pressure is generated by displacing silicone oil using a piston. An increase in pressure causes the ESs of sweet cherries to bulge outwards. The system pressure and height of bulging are measured using electronic pressure and displacement transducers, respectively. (B) The contour of the bulged ES was determined from a cross-section of an imprint and digitized. A range of geometrical models (spheroid, paraboloid and ellipsoid) were fitted to the contour.

A custom-made brass washer (12 mm inner diameter, 40 mm outer diameter, 3 mm thick) was mounted on the surface of a fruit in one of the two shoulder regions that border the cheek using a cyanacrylate adhesive (Loctite 406; Henkel AG & Co. KGaA/Loctite Deutschland GmbH, Munich, Germany). The fruit, with the washer attached, was placed in a sealed box above water (100 % relative humidity) and held for 10 min to accelerate curing of the adhesive. The ES was excised by cutting tangentially beneath the washer with a sharp razor blade. Because a cherry skin is naturally stressed *in vivo*, without the washer, this stress would normally be released very rapidly upon excision (Grimm *et al.* 2012). The washer procedure ensures that the *in vivo* stress is preserved in the excised ES (Knoche and Peschel 2006). The ES so obtained has the approximate shape of a ‘cap’ of a sphere with a maximum thickness in the middle of ∼2.4 ± 0.02 mm of an average fruit of 10.4 ± 0.1 g. The ES comprises cuticle, epidermis, hypodermis and adhering flesh tissue (Glenn and Poovaiah 1989). A washer with the ES attached was then mounted in the elastometer such that the cut surface (flesh) of the ES was oriented towards the silicone oil held in the body of the elastometer (Fig. 1A).

The elastometer consists of a chamber filled with silicone oil (Wacker AK10; Wacker Chemie AG, Munich, Germany). A motorized piston could be driven into the chamber, displacing the oil at a rate of 153 µl min^−1^. The displacement of the oil resulted in (i) an increase in pressure that was monitored with a pressure transducer (Typ 40PC100G; Honeywell International, Morristown, NY, USA) and (ii) the bulging of the 12-mm-diameter portion of the ES within the washer. The amount of bulging was quantified using a displacement transducer (KAP-S/5N; AST Angewandte System Technik GmbH, Wolnzach, Germany) of a material testing machine (BXC-FR2.5TN; Zwick GmbH & Co. KG, Ulm, Germany). The transducer probe (circular, flat, diameter 3 mm) was placed in contact with the skin surface at the centre of the ES and imposed a downward force on the skin of 0.05 N. The material testing machine was programmed to quantify the upward displacement (mm) caused by the bulging of the ES during the test. Unless otherwise stated, the pressure was increased continuously until the ES fractured. The recorded oil pressure in the elastometer at failure is referred to as the fracture pressure (*p*_fracture_, kPa), and the corresponding biaxial strain at failure is referred to as the fracture strain (*ɛ*_fracture_ mm^2^ mm^−2^). The static pressure in the system due to gravity was negligibly small. All experiments were performed under standard conditions at 22 °C.

*ɛ* and *ɛ*_fracture_ were calculated from the height of the bulging ES. A preliminary experiment was conducted to identify the geometry of the bulging ES. Castings (female) of several bulging ESs were prepared using a silicone casting material (Provil^®^ novo light regular; Heraeus Kulzer GmbH, Hanau, Germany). Subsequently, castings (male) were prepared from these, using hot-melt glue. The (male) castings were cross-sectioned and their contours were digitized using a binocular microscope system with image analysis (MZ10 F; Leica Microsystems GmbH, Wetzlar, Germany; Olympus DP71; Olympus Europa GmbH, Hamburg, Germany; software Cell-P; Olympus Soft Imaging Solution GmbH, Münster, Germany). Different geometrical models (e.g. spheroid, paraboloid and ellipsoid) were tested (Fig. 1B). The shape of the bulging ES was best described by the spheroid model as indexed by minimum deviance (M. Brüggenwirth, unpubl. res.). Therefore, all geometrical calculations were performed using this model. The surface area (*A*) of the bulging ES was calculated from the height (*h*) of the bulge and the inner radius of the washer (*R*) according to

$$A = (R^2 + h^2 ) \times \pi $$

The biaxial strain (*ɛ*) was then calculated as the increase in *A* (Δ*A*) as the ES bulged, relative to its initial surface area (*A*_0_):

$$\varepsilon = \displaystyle{{\Delta A} \over {A_0 }}$$

The modulus of elasticity (*E*) is a measure of the sample stiffness—a high *E* value implies a stiff ES requiring a high pressure for a small relative increase in area. Conversely, a low *E* is characteristic of an extensible ES where a low pressure causes a large increase in relative area. The *E* value was calculated from

$$E = \displaystyle{{\,p \times R^2 \times (R^2 + h^2 )} \over {h^3 \times t \times 2}}$$

where *p* is the pressure in the elastometer, *t* the thickness of the load-bearing skin layer (0.1 mm), *R* the radius of the orifice of the washer and *h* the height of the bulging ES. It is important to recognize that the strain in the bulging ES is not uniform but instead increases concentrically towards the centre (Chanliaud *et al.* 2002). Thus, the *E* calculated from the above equation is the apparent modulus averaged over the entire ES.

### Experiments

To compare data obtained in biaxial and uniaxial tensile tests of cherry skin and to quantify Poisson's ratio, both tests were conducted on the ES obtained from the same batch of ‘Regina’ fruit under standard conditions. Because it is technically impossible to maintain the *in vivo* strain of the skin when preparing skin segments for uniaxial tensile tests, we quantified the relaxation of the ES on excision by applying a 3 × 3 mm square pattern of four tiny white blobs of silicone adhesive (744 Silicone Adhesive/Sealant; Dow Corning^®^, Midland, MI, USA) before excising the ES. The dot pattern was photographed for image analysis. A dumbbell-shaped, biconcave specimen was then excised using a custom-made punch, such that the dot pattern was positioned in the waist region of the sample. The waist width of the ES was 4.25 mm, the maximum width 10 mm and the length 30 mm. Thickness averaged 2 mm. Preliminary experiments established that dumbbell-shaped ESs were needed to avoid failure of the ES at the clamps. The ESs were then mounted in a universal material testing machine (Z 0.5; Zwick/Roell, clamping distance *l*_0_ = 18 mm) equipped with a 10 N force transducer (KAP-Z; Zwick/Roell). The strain rate was 3 mm min^−1^. Calibrated digital photographs of the dot pattern were taken every 15 s (Canon EOS 550D, lens EFS 60 mm, f/2.8 Macro USM; Canon Deutschland GmbH, Krefeld, Germany). The distances between the dots in axial and transverse directions were quantified using image analysis (software Cell-P). The strains ($\varepsilon _{{\rm uniaxial}}^{{\rm axial}} $ and $\varepsilon _{{\rm uniaxial}}^{{\rm transverse}} $) were calculated from the increase in length (Δ*l*) relative to initial length (*l*_0_) according to

$$\varepsilon _{{\rm uniaxial}} = \displaystyle{{\Delta l} \over {l_0 }}$$

Poisson's ratio (*ν*) of cherry skin was calculated from

$$v = - \displaystyle{{\varepsilon _{{\rm uniaxial}}^{{\rm axial}} } \over {\varepsilon _{{\rm uniaxial}}^{{\rm transverse}} }}$$

Frequency distributions of *E*, *p*_fracture_ and *ɛ*_fracture_ were established by pooling control treatments of all experiments performed on the ES excised from mature field-grown ‘Regina’ fruit under standard conditions (22 °C).

To determine whether the elastometer can also be used for skins of other fruit crops, ESs from sour cherry, plum, grape and cape gooseberry were tested and their *E*, *p*_fracture_ and *ɛ*_fracture_ were determined as described above.

Potential relationships between the mechanical properties of ESs excised from the same fruit were studied in ‘Regina’. Two washers per fruit were mounted on the two shoulders of the fruit, and the ESs were then excised and tested as described above.

The consequence of releasing the *in vivo* strain of the skin when mounting the ES in the elastometer was investigated by comparing the *E*, *p*_fracture_ and *ɛ*_fracture_ of ESs (‘Samba’) that released or maintained their *in vivo* strain. For release of the *in vivo* strain, the ESs were excised (undercut) from the fruit using a razor blade and left on the fruit *in situ* to relax for 1 h to minimize drying. Because the half-time for strain relaxation is only 2.7 min (Grimm *et al.* 2012), the *in vivo* strain was considered to have been released by the end of the 1-h rest period. Thereafter, the brass washer was mounted on the relaxed ES and then transferred to the elastometer. These ESs were compared with those from the same batch of fruit in which the strain was maintained by mounting the washer prior to excision of the ES as described above. The amount of strain released from the relaxing ES was quantified in a separate experiment using the procedure described earlier (Grimm *et al.* 2012). Briefly, a square dot pattern of silicone sealant blobs (744 Silicone Adhesive/Sealant) was applied to the shoulder of the fruit. Digital photographs (Canon EOS 550D, lens EFS 60 mm, f/2.8 Macro USM) were taken before and immediately after excision of the ES, and thereafter at regular time intervals up to 48 h after excision. The areas enclosed by the dots were quantified (software Cell-P).

To identify potential anisotropy, the orientation of the ‘Regina’ ES in the washer was labelled. A square dot pattern (3 × 3 mm) of silicone sealant (744 Silicone Adhesive/Sealant) was applied to the centre of the ES. The ESs were then mounted in the elastometer. The pressure inside the chamber was increased stepwise and digital photographs of the dot pattern on the bulging ES were taken at each pressure step (Canon EOS 550D, lens EFS 60 mm, f/2.8 Macro USM). The distances between the dots in the longitudinal (parallel to the stylar scar, pedicel axis) and latitudinal directions (perpendicular to the stylar scar, pedicel axis) were quantified using image analysis (software Cell-P), corrected for curvature of the bulging segment, and the strains in the longitudinal and latitudinal directions were calculated according to

$$c = \displaystyle{{4h^2 + D^2 } \over {8h}}$$

$$b = 2 \times c \times \arcsin \left( {\displaystyle{k \over {2c}}} \right)$$

In these equations, *h* represents the height of the bulging ES, *D* the diameter of the orifice equivalent to the inner diameter of the washer, *c* the radius of the hypothetical sphere of which the ES bulging through the washer is a portion, *k* the distance between the dots and *b* the length of the arc.

From the longitudinal strain (*ɛ*_longitudinal_) and the latitudinal strain (*ɛ*_latitudinal_), the biaxial strain ($\varepsilon _{{\rm biaxial}}^{{\rm calculated}} $) was calculated according to

$$\varepsilon _{{\rm biaxial}}^{{\rm calculated}} = (\varepsilon _{{\rm longitudinal}} + 1) \times (\varepsilon _{{\rm latitudinal}} + 1) - 1$$

$\varepsilon _{{\rm biaxial}}^{{\rm calculated}} $ was then compared with the biaxial strain obtained from the height of the bulging ES as described above.

The effect of orifice diameter was investigated by mounting the ES excised from ‘Regina’ cherries in stainless steel washers of inner diameters of 6.4, 8.4, 10.5, 12 and 13 mm.

The effect of ES thickness was studied in field-grown ‘NY242’ fruit. Exocarp segments were cut to different thicknesses using spacers between the washer and the razor blade. Exocarp segments thinner than 1 mm were prepared by gently scraping away the flesh tissue using a sharpened scoop.

The roles of the cuticle, of the epidermal cell walls and of the hypodermal cell walls on the mechanical properties of the ES were examined in mature ‘Regina’ cherries. The cuticle was ground using fine sandpaper (K800; EMIL LUX GmbH & Co. KG, Wermelskirchen, Germany) and the treatment effect was documented by fluorescence microscopy (MZ10 F; Leica Microsystems GmbH, GFP plus filter module with 480/40 nm excitation wavelength, emission wavelength ≥510 nm) following infiltration of the ES using a 0.1 % acridine orange solution for 10 min. To address the role of cell walls of the tissue underlying the cuticle and to mimic the process of fruit softening, ESs mounted in washers were incubated in a 10 % v/v solution containing pectinase (Panzym Super E; Novozymes A/S, Bagsvaerd, Denmark). The pH of the non-buffered solution was pH 5.1 at the beginning and pH 5.0 at the end of the 3-h incubation period at 22 °C. Thereafter, the ESs were removed from the enzyme solution and tested in the elastometer as described above.

The total strain of a bulging ES (‘Merchant’) was partitioned into elastic, viscoelastic and plastic strains. The ESs were mounted as described above and subjected to a creep-relaxation test, comprising ‘loading’, ‘holding’ and ‘unloading’ phases. First, during the loading phase, the pressure was increased to 10 kPa. Next, in the holding phase, the pressure was held constant for 10 min allowing the ES to creep. Last, in the unloading phase, the pressure was suddenly decreased to 0 kPa, allowing the ES to relax. The instantaneous strain during the loading phase is referred to as the elastic strain. The total strain at the end of the holding phase is equal to the sum of the viscoelastic and the plastic strains (in the literature it is sometimes referred to as the ‘creep strain’). The strain that remains after the relaxation phase is the plastic strain.

### Data analysis and presentation

Data analysis was limited to those ESs that did not fracture at the edge of the orifice. Fracture at the edge of the ESs may have resulted from a mounting artefact or a skin flaw that was not noticed in a visual inspection. The majority of ESs failed in the centre. The fraction that failed at the edge amounted to ∼20 % of the population of ESs investigated. These results were excluded from the analysis. Unless otherwise stated, the ES data presented are the means ± standard errors of the means (SEM). Following analysis of variance, mean comparisons were performed using Tukey's studentized range test (*P* ≤ 0.05, packet multcomp 1.2–12, procedure glht, R 2.13.1; R Foundation for Statistical Computing, Wien, Austria) and the *t*-test (*P* ≤ 0.05, R 2.13.1). The significance of coefficients of correlation (*r*) and determination (*r*^2^) for probabilities (*P*) of 0.05, 0.01 and 0.001 are indicated by *, ** and ***, respectively.

## Results

**Figure 2.**
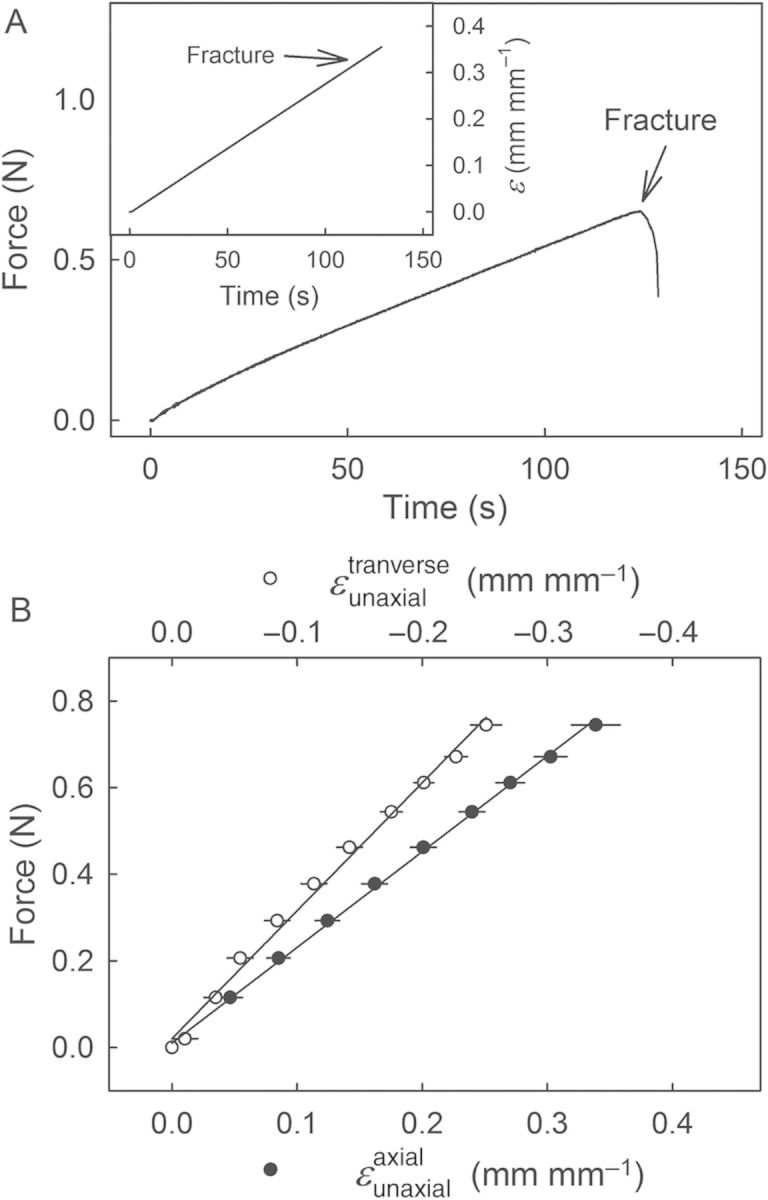
Uniaxial tensile test of a biconcave, dumbbell-shaped, exocarp strip carved from sweet cherry skin. (A) Representative time course of force and strain (*ɛ*; inset) until fracture. (B) Relationship between force and the strains in the directions of the applied force (axial strain; $\varepsilon _{{\rm uniaxial}}^{{\rm axial}} $) and perpendicular to the applied force (transverse strain; $\varepsilon _{{\rm uniaxial}}^{{\rm transverse}} $). Note that the negative $\varepsilon _{{\rm uniaxial}}^{{\rm transverse}} $ results from the marked narrowing of the strip when subjected to a uniaxial load. Data represent means ± SEM (*n* = 10).

Performing uniaxial tensile tests using dumbbell-shaped, biconcave ES specimens resulted in linear increases in force and axial strain vs. testing time, to the point of fracture (Fig. 2A). The corresponding force vs. (axial) strain diagrams were also linear (*r*^2^ = 0.99***; Fig. 2B). As the ES extended in the axial direction, considerable narrowing was observed, perpendicular to the applied force. Calculating the corresponding $\varepsilon _{{\rm uniaxial}}^{{\rm transverse}} $ revealed that this $\varepsilon _{{\rm uniaxial}}^{{\rm transverse}} $ was linearly and negatively related to the axial force (*r*^2^ = 0.99***; Fig. 2B). The Poisson's ratio calculated from the slopes of the force vs. $\varepsilon _{{\rm uniaxial}}^{{\rm axial}} $ and force vs. $\varepsilon _{{\rm uniaxial}}^{{\rm transverse}} $ relationships was 0.74 ± 0.03.

**Figure 3.**
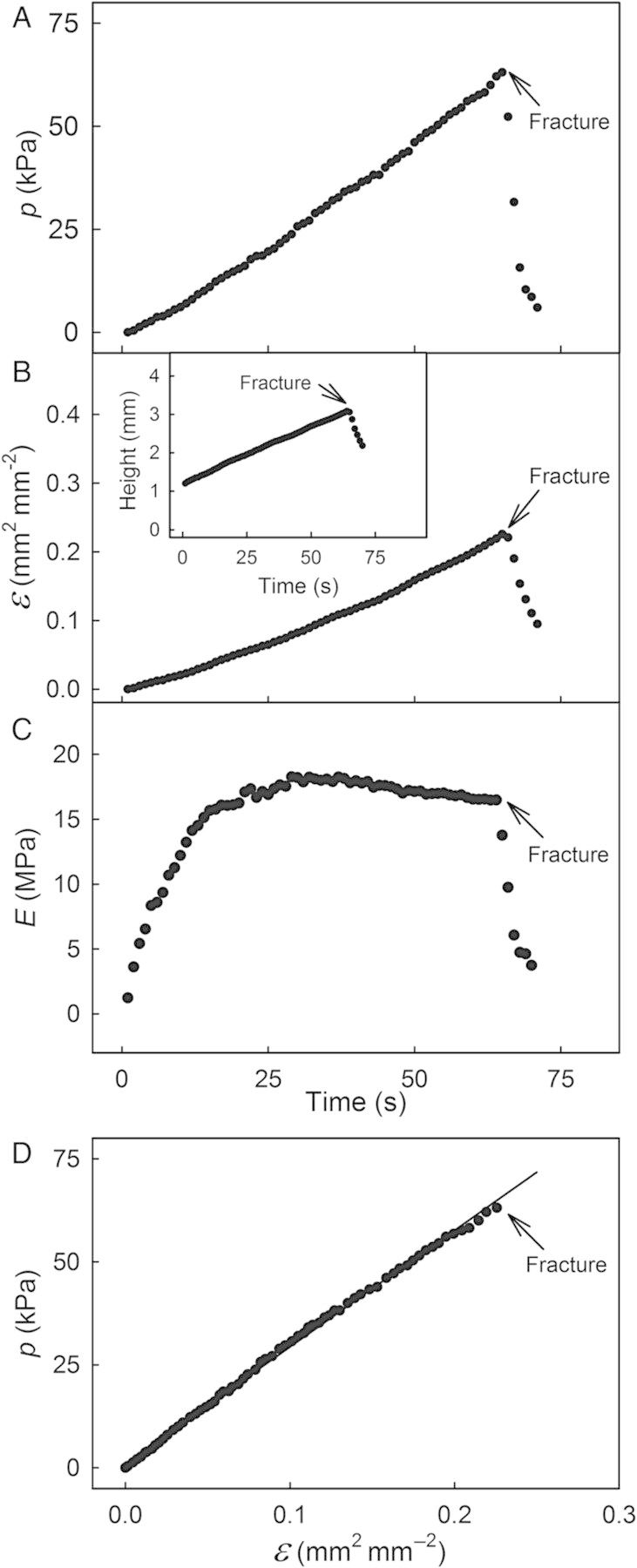
Representative time courses of the change in pressure (*p*; A), in strain (*ɛ*; B), in height of the bulging ES of sweet cherry fruit (B, inset), and in the modulus of elasticity (*E*; C) during a biaxial tensile test. The last six data points represent points recorded after fracture. (D) Pressure/strain diagram of a representative ES until fracture.

When displacing silicone oil by driving the piston into the chamber of the elastometer, *p* increased linearly with time, causing the ES to bulge as indicated by an essentially linear increase in height (Fig. 3A and B). The *ɛ* value calculated therefrom also increased with time. The modulus *E* increased rapidly to a maximum of 18 MPa at ∼25 s after initiation of the test and was then approximately constant until failure (Fig. 3C). When the ES failed, *p*, *ɛ*, and *E* decreased instantaneously. The pressure–strain diagrams were essentially linear up to the point of fracture (*r*^2^ = 0.98***; see Fig. 3D for a representative ES).

**Figure 4.**
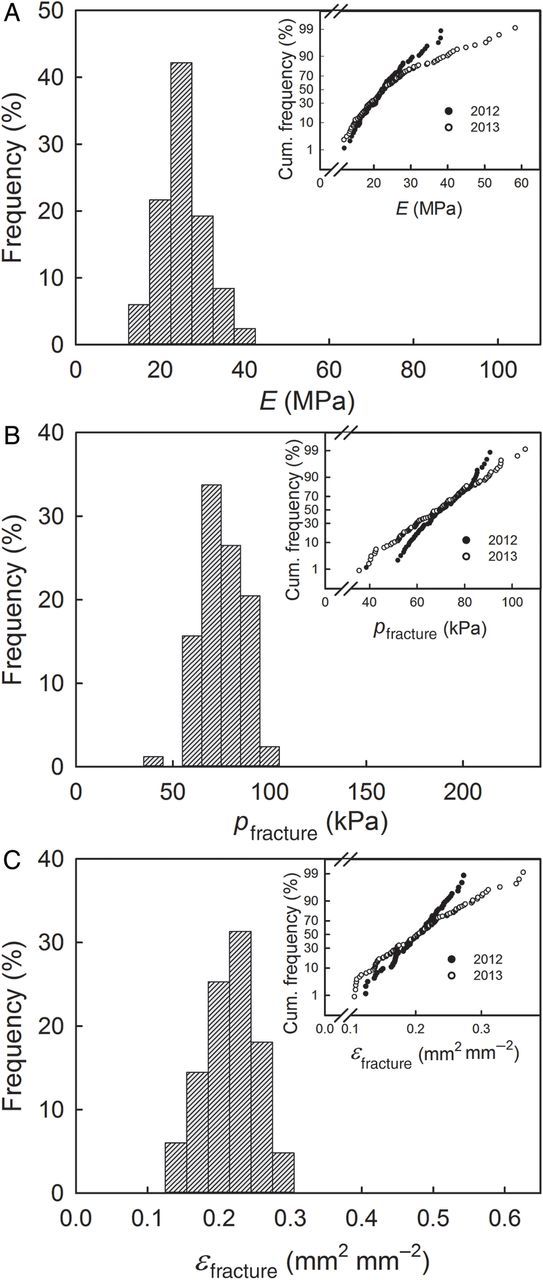
Frequency distributions of the modulus of elasticity (*E*; A), the pressure at fracture (*p*_fracture_; B) and the strain at fracture (*ɛ*_fracture_; C) of ESs of sweet cherry fruit in 2012. Insets: probability plots of cumulative frequency distributions of *E*, *p*_fracture_ and *ɛ*_fracture_ of the same cultivar in 2012 (*n* = 84) and 2013 (*n* = 115).

Frequency distributions of *E*, *p*_fracture_ and *ɛ*_fracture_ were approximately symmetrical and the corresponding normal probability plots were linear, indicating that *E*, *p*_fracture_ and *ɛ*_fracture_ followed a normal distribution (Fig. 4). There was little difference between the means and medians of *E*, *p*_fracture_ and *ɛ*_fracture_ for the ES of fruit from the 2012 and 2013 seasons. However, the variability was somewhat larger in 2013 as indicated by the lower slope of the normal probability plots and the larger standard errors, coefficients of variations and ranges (Table 1).

**Table 1.** Means, medians, standard errors of means (SEM), coefficients of variation (CV) and ranges of modulus of elasticity (*E*), fracture pressure (*p*_fracture_) and fracture strain (*ɛ*_fracture_) in ESs excised from sweet cherries and subjected to a biaxial tensile test. Exocarp segments were prepared from the cheek of mature fruit in the 2012 (*n* = 84) and 2013 (*n* = 115) growing seasons.

	Year	Mean	Median	SEM	CV	Range	
						Min.	Max.
*E* (MPa)	2012	23.2	22.2	0.66	0.25	12.0	41.2
	2013	25.2	22.7	0.94	0.40	10.5	60.3
*p*_fracture_ (kPa)	2012	70.8	70.0	1.2	0.15	38.6	92.6
	2013	69.0	69.6	1.4	0.22	35.6	107.6
*ɛ*_fracture_ (mm^2^ mm^−2^)	2012	0.20	0.20	0.01	0.18	0.12	0.34
	2013	0.21	0.20	0.01	0.29	0.11	0.38

Qualitatively similar data as in sweet cherry were obtained for sour cherries, plums, grapes and cape gooseberries, but the range in mechanical properties was somewhat larger (Table 2). The lowest *E* was measured for the sour cherry cultivar ‘Ungarische Traubige’, and the highest for the ‘Hanita’ plum. The maximum *p*_fracture_ was measured in the ‘Fanny’ grape berry, and the highest *ɛ*_fracture_ in the sour cherry cultivar ‘Ungarische Traubige’ (Table 2).

**Table 2.** Modulus of elasticity (*E*), fracture pressure (*p*_fracture_) and fracture strain (*ɛ*_fracture_) of ESs excised from the equatorial region of mature fruit of sour cherry, European plum, grape and cape gooseberry. Data are means and standard errors of means of 25 replications per species.

Species	Cultivar	*E* ± SE (MPa)	*p*_fracture_ ± SE (kPa)	*ɛ*_fracture_ ± SE (mm^2^ mm^−2^)
Sour cherry	Morellenfeuer	5.9 ± 0.2	27.8 ± 0.8	0.30 ± 0.01
	Ungarische Traubige	3.5 ± 0.2	36.2 ± 1.1	0.53 ± 0.01
European plum	Hanita	35.9 ± 3.2	53.1 ± 4.2	0.15 ± 0.01
	Wangenheim	25.4 ± 7.8	65.8 ± 3.4	0.18 ± 0.01
	Nancy	27.3 ± 1.9	56.8 ± 3.0	0.18 ± 0.01
Grape berry	Fanny	21.5 ± 1.6	78.9 ± 2.5	0.23 ± 0.02
	Nero	20.8 ± 1.7	75.0 ± 2.8	0.19 ± 0.01
Cape gooseberry	Unknown	20.5 ± 0.8	94.7 ± 3.9	0.25 ± 0.01

**Figure 5.**
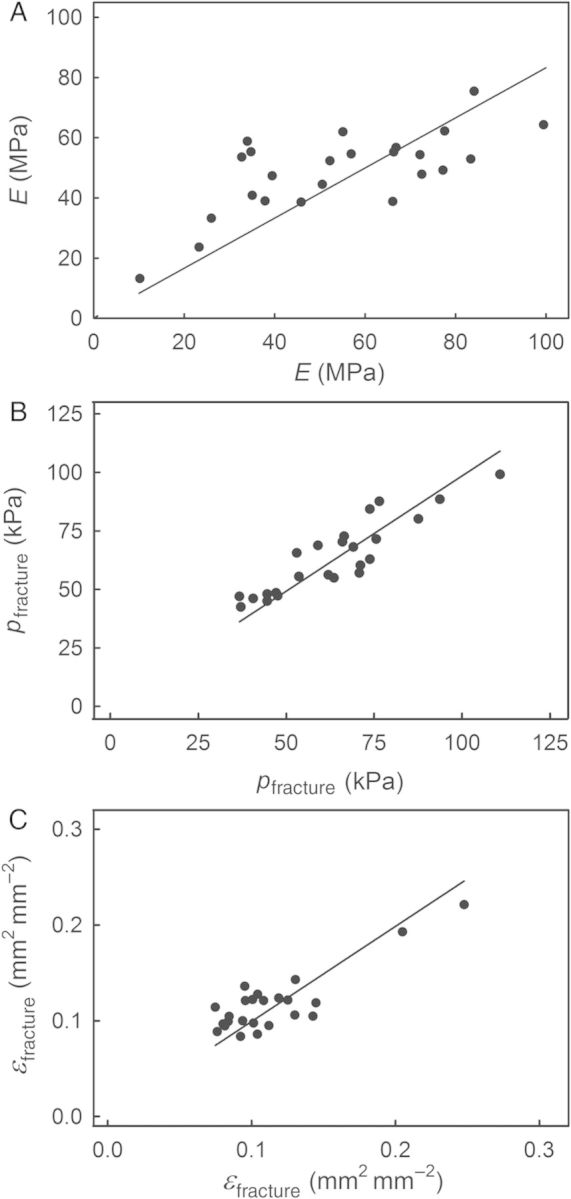
Relationship between the modulus of elasticity (*E*; A), the pressures at fracture (*p*_fracture_; B) and the strain at fracture (*ɛ*_fracture_; C) of pairs of ESs excised from opposite shoulders of the same sweet cherry fruit. Data represent means ± SEM (*n* = 24).

The *E*, *p*_fracture_ and *ɛ*_fracture_ of pairs of ESs excised from opposite sides of the same fruit were significantly correlated, with coefficients of correlation of *r* = 0.73***, 0.74*** and 0.54**, respectively (Fig. 5).

Upon excision, the ES relaxed and decreased in surface area, indicating the presence of significant strain *in vivo*. For the batch of fruit used in this experiment, the *in vivo* strains averaged 15.5 ± 1.4 %, which is somewhat lower than the strains reported by Grimm *et al.* (2012; range 18.7–36.0 %). The presence of *in vivo* strain significantly affected the mechanical properties of the ES (Table 3). Exocarp segments that released the *in vivo* strain and relaxed before the biaxial tensile test had higher *ɛ*_fracture_ and lower *E* than those ESs where the *in vivo* strain was maintained (Table 3). There were no significant differences in *p*_fracture_ between ESs with and without *in vivo* strain (Table 3).

**Table 3.** Effect of maintaining or releasing the *in vivo* strain and stress of ESs excised from mature sweet cherries on the modulus of elasticity (*E*), fracture pressure (*p*_fracture_) and fracture strain (*ɛ*_fracture_). *Means within columns followed by the same letter are not significantly different, *t*-test. *P* < 0.05. Values are means and standard errors of means of 10 replicates.

*In vivo* strain	*E* (MPa)	*p*_fracture_ (kPa)	*ɛ*_fracture_ (mm^2^ mm^−2^)
Maintained	16.6 ± 1.2a*	51.0 ± 1.6a	0.21 ± 0.01a
Released	11.4 ± 1.0b	50.6 ± 1.6a	0.29 ± 0.01b

**Figure 6.**
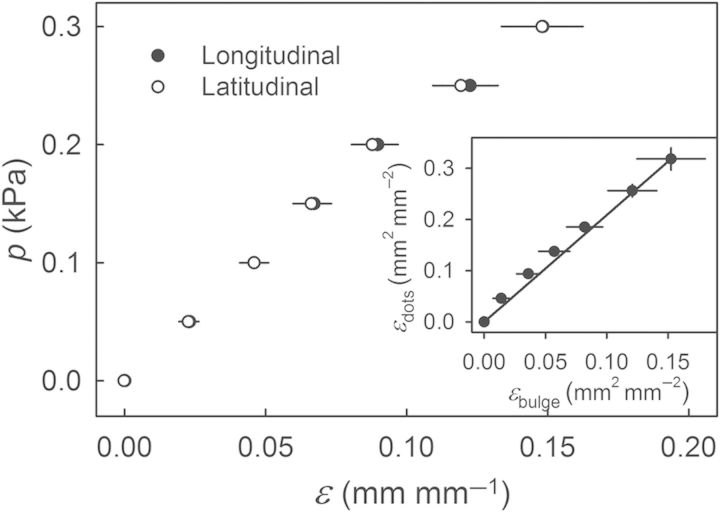
Relationship between pressure (*p*) and longitudinal or latitudinal strains (*ɛ*) of ESs excised from sweet cherry. The longitudinal strain is that in the direction of the stylar scar/pedicel axis, and the latitudinal strain is perpendicular to it. The two strains were measured using a square pattern of dots applied to the ES. Inset: relationship between the biaxial strain calculated from the height of the bulging ES and the biaxial strain measured using the dot pattern. The regression line has a slope of 2.16 ± 0.06 (*r*^2^ = 0.99***). Data represent means ± SEM (*n* = 10).

The *ɛ*_latitudinal_ and *ɛ*_longitudinal_ on the bulging ES were essentially identical, indicating that the cherry skin was isotropic in the tangential plane (Fig. 6). The $\varepsilon _{{\rm biaxial}}^{{\rm calculated}} $ measured from the dot pattern applied to the centre of the ES increased linearly as the pressure increased (data not shown) and was linearly related to the biaxial strain obtained from the height of the bulging ES (*r*^2^ = 0.97***). It should be noted that the biaxial strain derived from the dot pattern in the centre of the ES was always larger by a factor of 2.16 ± 0.06 (*r*^2^ = 0.99***) than the mean strain obtained from the height of the bulging ES (Fig. 6, inset).

**Figure 7.**
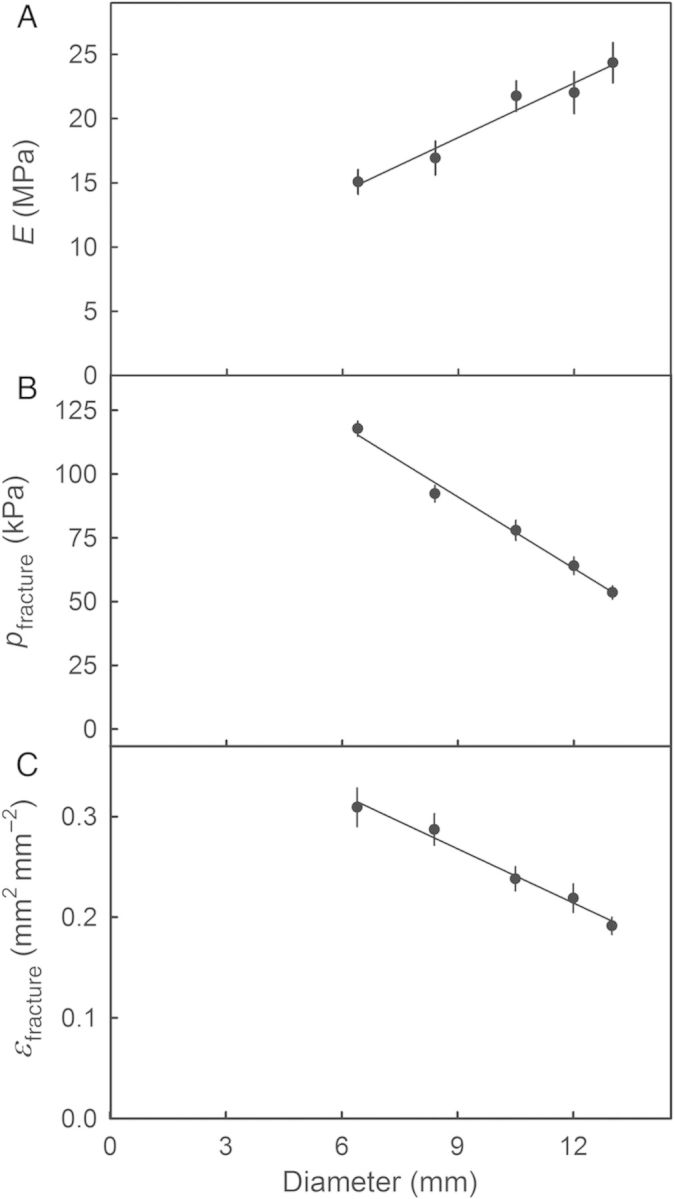
Effect of diameter of ESs excised from sweet cherry fruit on the modulus of elasticity (*E*; A), pressure at fracture (*p*_fracture_; B) and the strain at fracture (*ɛ*_fracture_; C). Data represent means ± SEM (*n* = 20).

The value of *E* increased linearly as the orifice diameter increased, suggesting that ESs were stiffer when mounted in a larger orifice (Fig. 7). However, *p*_fracture_ and *ɛ*_fracture_ decreased as the orifice diameter increased (Fig. 7).

**Figure 8.**
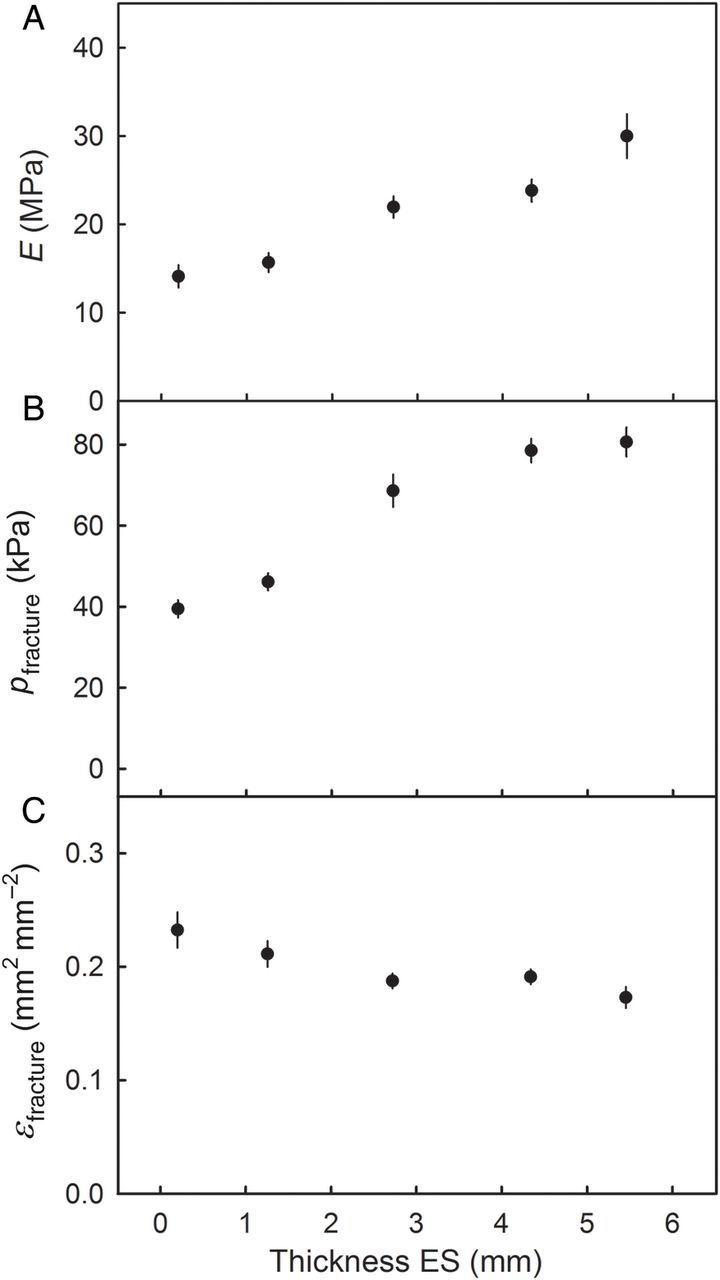
Effect of thickness of ESs excised from sweet cherry skin on the modulus of elasticity (*E*; A), the pressure at fracture (*p*_fracture_; B) and the strain at fracture (*ɛ*_fracture_; C). Data represent means ± SEM (*n* = 20).

The mechanical properties of ESs also depended on their thickness. Increasing thickness also caused the values of *E* and *p*_fracture_ to increase but that of *ɛ*_fracture_ decreased (Fig. 8).

Enzyme digestion of the cell walls of epidermal and hypodermal cells decreased *p*_fracture_ and *ɛ*_fracture_, whereas abrading the cuticle decreased only *ɛ*_fracture_ (Table 4). Neither treatment had any effect on *E*.

**Table 4.** Effect of abrading the cuticle and of digesting cell walls of epidermal and hypodermal cells of ESs excised from the cheek of mature sweet cherries on the modulus of elasticity (*E*), fracture pressure (*p*_fracture_) and fracture strain (*ɛ*_fracture_). The cuticle was abraded using sandpaper. In a further treatment, the support of the cuticle by epidermal and hypodermal cell layers was weakened by digesting the cell walls of the epidermal and hypodermal cell layers using pectinase. Untreated ES served as control. For details see the Methods section. *Means separation by Tukey's studentized range test, *P* < 0.05. Values are means and standard errors of means.

Treatment	*N*	*E* (MPa)	*p*_fracture_ (kPa)	*ɛ*_fracture_ (mm^2^ mm^−2^)
Control	18	13.6 ± 1.0a*	44 ± 2.3a	0.20 ± 0.01a
Abraded cuticle	19	16.8 ± 1.5a	41 ± 2.4a	0.17 ± 0.01b
Digested epidermis and hypodermis	22	14.1 ± 1.1a	26 ± 2.0b	0.16 ± 0.01b

**Figure 9.**
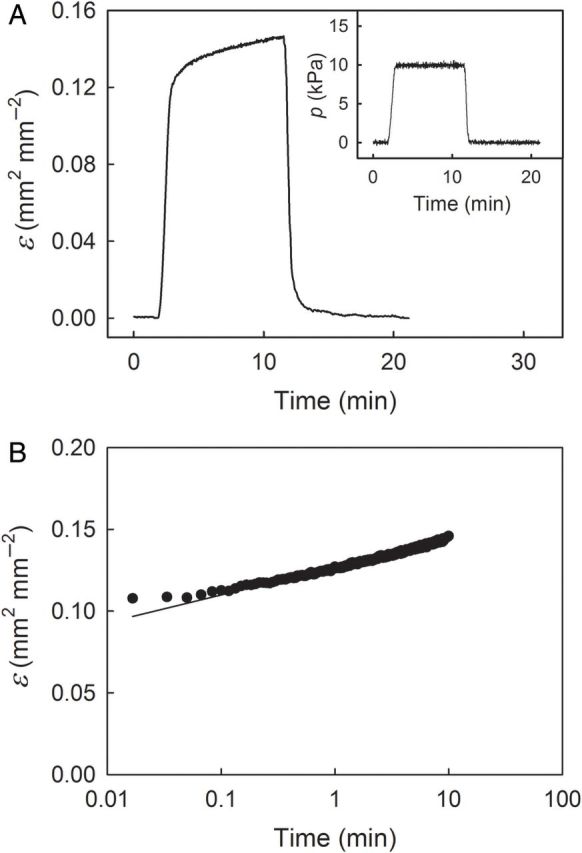
Representative time course of strain (*ɛ*; A) and pressure (*p*; A, inset) of an ES excised from sweet cherry skin during a biaxial, creep-relaxation test. The pressure was increased during the initial loading phase, held constant during the holding phase and decreased during the subsequent unloading phase. (B) Strain during the holding phase is redrawn on a log-transformed timescale.

Increasing *p* during the loading phase of the creep-relaxation test caused an increase in instantanous elastic strain (Fig. 9A). When *p* was held constant during the subsequent holding phase, the ES extended (crept) due to viscoelastic strain. Upon unloading, the ES relaxed, indicating a release of elastic and viscoelastic strains. No irreversible, plastic strain was detected. Plotting viscoelastic strain during the holding period vs. log-transformed time yielded a linear relationship (*r*² = 0.98***; Fig. 9B).

## Discussion

Our data demonstrate that (1) the elastometer allows a reproducible *in vitro* test of cherry skin; (2) the cherry skin is isotropic in the tangential plane and shows both elastic and viscoelastic strain; (3) the epidermis and hypodermis represent the structural ‘backbone’ of cherry skin. The contribution of the cuticle to the skin's mechanical properties is minimal. These findings are discussed in detail below.

An *in vitro* test system simulating growth stresses must meet the following conditions. (i) Skin samples must maintain their key mechanical properties when excised and mounted in the testing device. (ii) The test strains imposed on the ES *in vitro* must operate in the same directions as the ‘natural’ ones *in vivo*. (iii) All strains must be applied in a defined manner. The elastometer used in our study met all three requirements.

First, the *in vivo* strain was maintained in our tests by attaching a washer to the ES area of the fruit before excision. This procedure essentially fixes and maintains the *in vivo* strain in the excised ES (Knoche and Peschel 2006). Indeed, if strain were not fixed, and the ES had relaxed upon excision, then *ɛ*_fracture_ would increase and *E* would decrease, whereas *p*_fracture_ would not be affected (Table 3). Also, silicone oil was used to pressurize the elastometer, not water. Compared with water, silicone oil is much less likely to modify tissue water potential (water uptake, cell bursting etc.). If water were to be used, osmotic buffering probably would be required to avoid modifying tissue water relations.

Second, both the biaxial tensile test by Bargel *et al.* (2004) and our elastometer subject skin samples to biaxial tangential strains, thereby simulating the distribution of growth strains in spherical fruit *in vivo*. Because the orifice of the washer is circular, the force vectors are expected to be uniform at least in the centre of the bulging ES where most ESs failed. It may be argued that biaxial *ɛ*_fracture_ can also be calculated from *ɛ*_fracture_ determined in uniaxial tests. However, performing the calculations clearly demonstrates that this would result in a four-fold overestimation of *ɛ*_fracture_ (79 ± 3.9% vs. 20 ± 0.6 % for the calculated biaxial vs. the true *ɛ*_fracture_; Table 1).

Third, the elastometer allowed the ES to be stressed in a defined manner, i.e. using a pressurized fluid to strain the ES at a constant and defined rate up to some preset level or by performing creep-relaxation tests with and without cyclic application of pressure (M. Brüggenwirth, unpubl. res.). It is important to recognize that the application of uniform pressure does not result in uniform strain across the ES. In a bulging, circular specimen, a strain gradient exists from a high value in the centre to a lower one at the edge (Chanliaud *et al.* 2002). Further, the strain at the edge is uniaxial. A strain gradient was also present in our ES, where the strain calculated from the dot pattern in the centre was twice the mean strain calculated from the height of the bulging ES (Fig. 6, inset). The decrease of *p*_fracture_ and *ɛ*_fracture_ observed when the washer orifice diameter was increased is also accounted for by a gradient in strain. The probability of failure increases because strains in the centre of the bulging ES of larger diameter would be greater.

The elastometer allows reproducible testing of the ES of fleshy fruit such as cherries under defined conditions. Reproducibility is indicated by the significant correlation of evaluations of *E*, *p*_fracture_ and *ɛ*_fracture_ in pairwise comparisons where these mechanical properties were tested on pairs of ESs excised from the two shoulders of the same fruit (Fig. 5). Also, frequency distributions of the mechanical properties were very similar for fruit from the 2012 and 2013 seasons. The normal probability plots were largely linear, indicating symmetrical distributions with coefficients of variation ranging from 0.15 to 0.40. These values are not unusual for biological materials that are well known to be highly variable compared with non-biological ones (Table 1). Thus, the elastometer proved to be a useful tool for standardized testing of fruit skins.

In cherries, preferential orientation of epidermal cells has been reported for the cheek and neighbouring shoulder region (Peschel and Knoche 2005). This, however, had no effect on isotropy, for the skin of the shoulder regions was found to be isotropic in the tangential plane, with *ɛ*_longitudinal_ and *ɛ*_latitudinal_ being indistinguishable (Fig. 6).

Based on the following observations, cherry skin behaves like a viscoelastic composite. (i) During the holding phase of the creep-relaxation experiments, strain increased continuously. This ‘creep’ strain during the holding phase must have been primarily viscoelastic because there was essentially no irreversible (i.e. ‘plastic’) strain detectable during the unloading phase (Fig. 9). (ii) Plotting the strain increase during the holding phase vs. a logarithmic timescale yields a linear relationship: a characteristic of viscoelastic materials (Cosgrove 1993). Viscoelasticity of cherry skin is also reported in Grimm *et al.* (2012) and is typical of the skins of other soft-fruit species such as grape (Hankinson *et al.* 1977) and tomato (Petracek and Bukovac 1995; Matas *et al.* 2004). Moreover, it is a characteristic of cell walls in general (Cosgrove 1993) being primarily related to hemicelluloses (Chanliaud *et al.* 2002) and is typical for the deformation of turgid cells (Niklas and Spatz 2012).

The elastic strain component, which makes up an even larger contribution to total strain than does viscoelastic strain, also originates from the cell walls (Jackman *et al.* 1992) and particularly from the cellulosic fraction (Chanliaud *et al.* 2002). At the loads applied in our experiments, a plastic strain was not detectable (Fig. 9). In this respect, cherry skins may differ from tomato and grape skins where irreversible, plastic components of deformation have been identified (Lang and Düring 1990; Petracek and Bukovac 1995; Matas *et al.* 2004).

The pressure/strain diagrams obtained in our experiments were essentially linear, which contrasts with Bargel *et al.* (2004) who report rising slopes in their pressure/displacement diagrams (e.g. see Fig. 5 in Bargel *et al.* 2004). This observation is interpreted as a ‘strain hardening’ of the fruit skin (Bargel *et al.* 2004). However, an alternative explanation is that those samples released their *in vivo* strain immediately following excision and that the subsequent increases in the slope of their pressure/displacement diagrams were the result of a re-establishment of the *in vivo* strain that had been present prior to excision. Grimm *et al.* (2012) demonstrated that cherry skin is significantly strained (18.7–36 % depending on cultivar) and that this *in vivo* strain is released very rapidly upon excision (half-time = 2.7 min). This interpretation would also account in part for the markedly higher *ɛ*_fracture_ of 90 % that was observed by Bargel *et al.* (2004; recalculated from their Fig. 6). However, when simulating the release of strain in our elastometer, the difference in *ɛ*_fracture_ between ESs that maintained or released their *in vivo* strain was smaller, indicating that other factors may also be involved. The release of strain had no effect on *p*_fracture_. The range of *p*_fracture_ in our study (35–107 kPa; Table 1) is similar to that in Bargel *et al.* (2004; range 40–110 kPa). No differences in *p*_fracture_ were observed either with or without *in vivo* strain (Table 3).

We suggest that the mechanical properties of the ES are dominated by those of the epidermis and hypodermis and *not* by those of the cuticle. This hypothesis is supported by the following observations.

(i) Fruit softening, simulated by incubation in pectinase, reduced both *E* and *ɛ*_fracture_ by ∼30 % (Table 4). Consistent with this is the earlier observation that complete skin failure occurred in ESs that had their *in vivo* strain fixed (using a washer) and that were incubated with the washer attached in cellulase and pectinase (Knoche and Peschel 2006). During incubation, the enzymes weakened the cellular support of the cuticular membrane (CM). The subsequently occurring failure of the CM indicates that the CM is quite unable to sustain the degree of strain existing *in vivo* without the mechanical support offered by the underlying cell layers (Knoche and Peschel 2006).

(ii) Abrasion of the cuticle had only a small effect on the mechanical behaviour of the ES (Table 4). The slight decrease in strain observed here may have resulted from minor damage to some of the epidermal cell walls during abrasion. Also, Knoche and Peschel (2006) and Grimm *et al.* (2012) further demonstrate that the mechanical contribution of the cuticle in strain release is small and negligible. These observations are not surprising considering the delicate nature of the *c*. 1-µm-thick cuticle (Knoche and Peschel 2006) relative to the *c*. 85-µm-thick cellular component of the skin composite (i.e. the epidermis + hypodermis; Glenn and Poovaiah 1989). Also, considering the common presence of CM microcracks (Peschel and Knoche 2005) vs. the obvious, load-bearing properties of the collenchymatous (thickened) epidermal and hypodermal cells. Moreover, in apple, pear and tomato that have even thicker CM (*c*. 28.2, 17.3 and 15.5 g m^−2^ equivalent to a calculated mean thickness of 23.3, 14.3 and 12.8 µm for apple, pear and tomato, respectively; Khanal *et al.* 2013*a, b*) it is the cell layers of the skin that seem to support its mechanical stability (Matas *et al.* 2004; Khanal *et al.* 2013*a*; Khanal and Knoche 2014).

(iii) We observed little effect of varying the thickness of the ES (from 0.2 to 5.5 mm) on its mechanical properties. Noting that our technique varies only the depth of the adhering flesh cells, we can infer that these cells contribute very little to the skin's mechanical properties (Fig. 8). We reasonably conclude that the mechanical properties of the skin reside in the *c*. 85-µm-thick epidermal and hypodermal cell layers and are not significantly affected by either the CM or the flesh cells. Incidentally, we note that the cells in these layers have much smaller lumina and thicker walls than the flesh cells (Glenn and Poovaiah 1989).

## Conclusions

Our experiments demonstrate that the elastometer is a useful instrument that allows strained skin samples to be tested *in vitro* under conditions that simulate those likely to be found *in vivo*. Using this system it is now possible to quantify and establish the basis of the fundamental mechanical properties of fruit skins. The data presented here indicate that the cherry skin is isotropic in the tangential plane and exhibits both elastic and viscoelastic behaviour. It has further been established that the epidermis and hypodermis (but not the cuticle or flesh) represent the structural ‘backbone’ of cherry skin. These findings have important consequences, including a new understanding of the mechanism of skin failure *in vivo*. Viscoelastic deformation decreases skin stress (i.e. limits stress buildup with growth) and also prevents the development of significant tissue pressure inside the fruit. Based on current theories for cracking, this will decrease the likelihood of skin failure.

## Sources of Funding

This study was supported in part by a grant from the Deutsche Forschungsgemeinschaft (KN402/8-1).

## Contributions by the Authors

M.K. obtained the funds to support the study. H.F. conducted preliminary experiments to establish the test system. M.K., H.F. and M.B. designed the experiments. M.B. conducted the experiments. M.K. and M.B. analysed the data and wrote the manuscript. M.K., H.F. and M.B. revised and edited the paper.

## Conflicts of Interest Statement

None declared.
